# Supplementary material for: Hyperuricemia during Pregnancy Leads to a Preeclampsia-Like Phenotype in Mice
Source: Cells. 2022 Nov 21;11(22):3703. doi: 10.3390/cells11223703 (PMC9688737; doi:10.3390/cells11223703)
Supplement: Supplementary file 1 [file cells-11-03703-s001.zip › cells-1977784-supplementary.pdf]

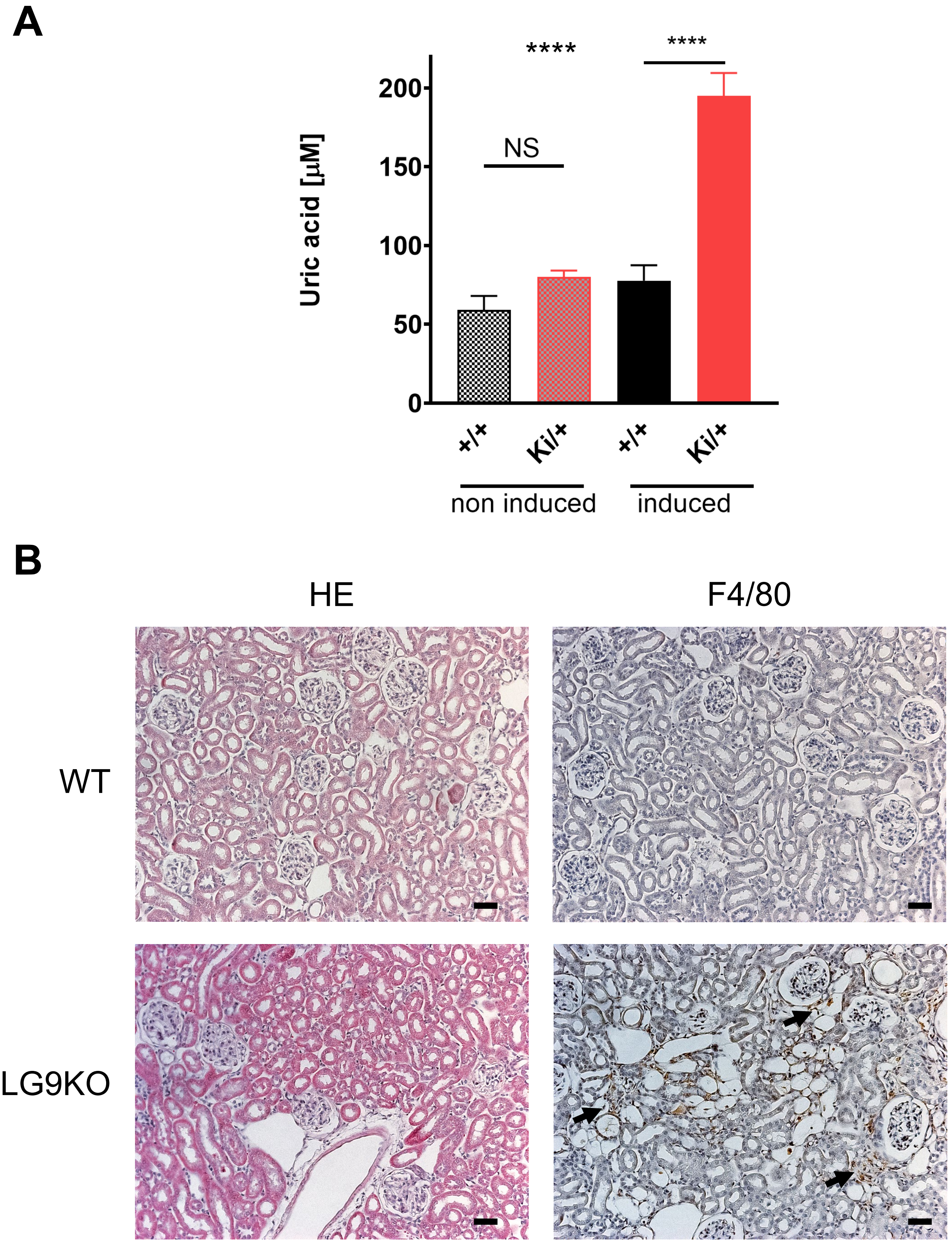

Figure S1. Uric acid serum levels and renal injuries: (A) Uric acid concentration of LG9KO (+/ki) and WT (+/+) animals before and after Tamoxifen induction. (B) HE staining and F4/80 staining of WT and LG9KO animals from kidney obtained after blood pressure measurements. Hyperuricemia leads to invasion of macrophages into the kidney Scale bar shows 25μm.

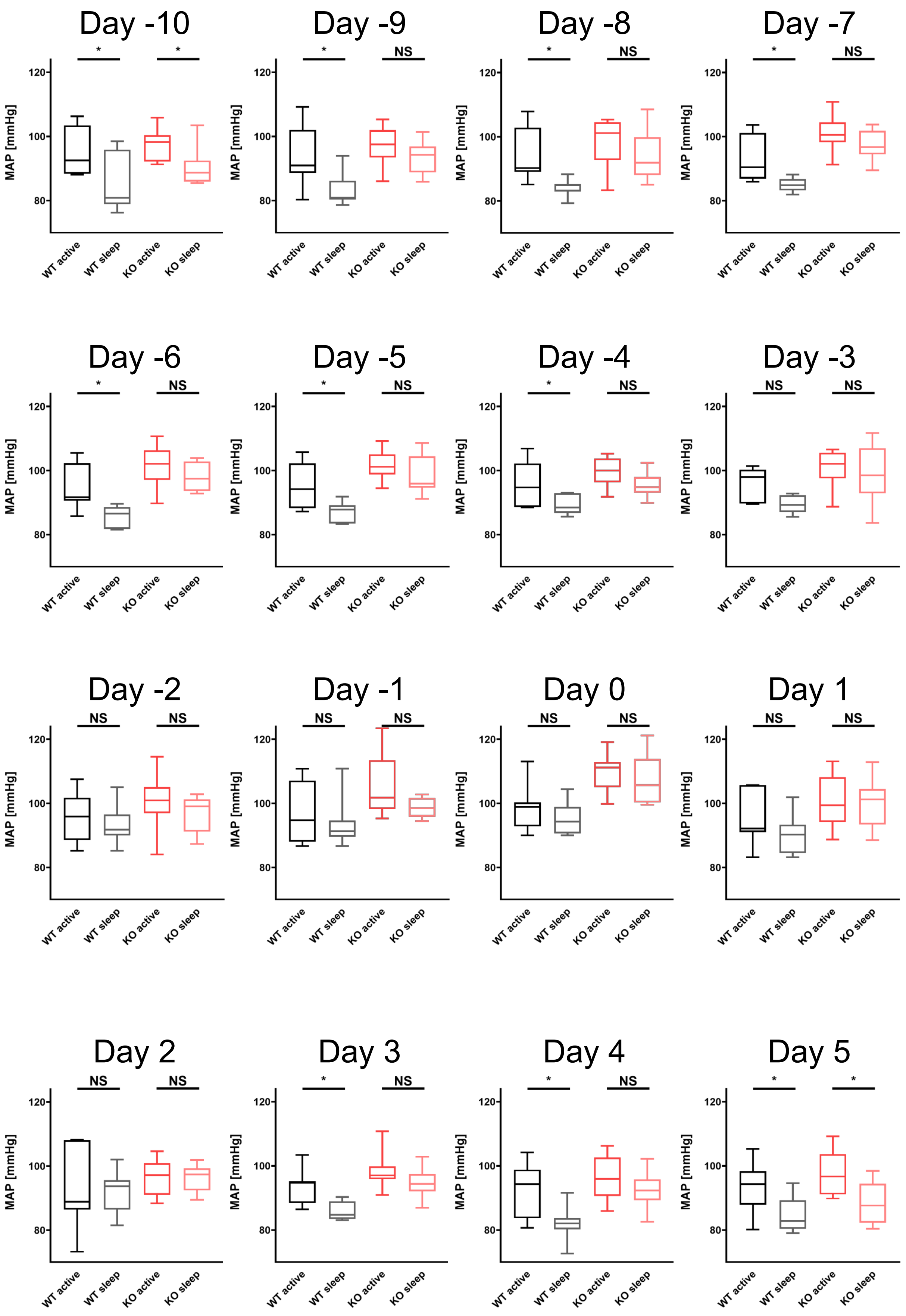

Figure S2. Time course of circadian blood pressure dipping pattern (Day -10 – Day 5): Dipping analysis of WT (black) and LG9KO (red) animals from day -10 to day 5. Day 0 is defined as the day of giving birth.

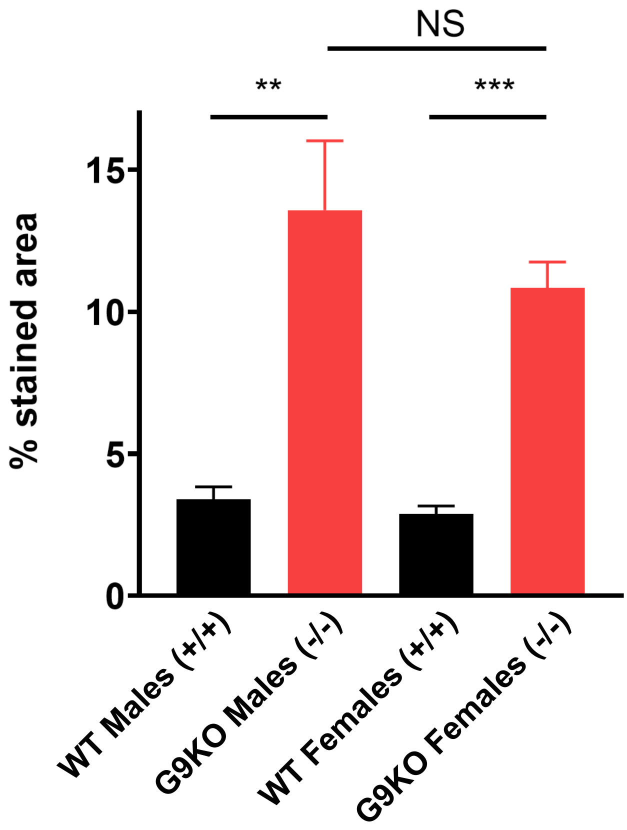

Figure S3. Macrophage invasion. Assessment of macrophage invasion of G9KO males and females compared to WT males and females from kidneys obtained on day 70. Male and female G9KO animals show massive increase of macrophage invasion.

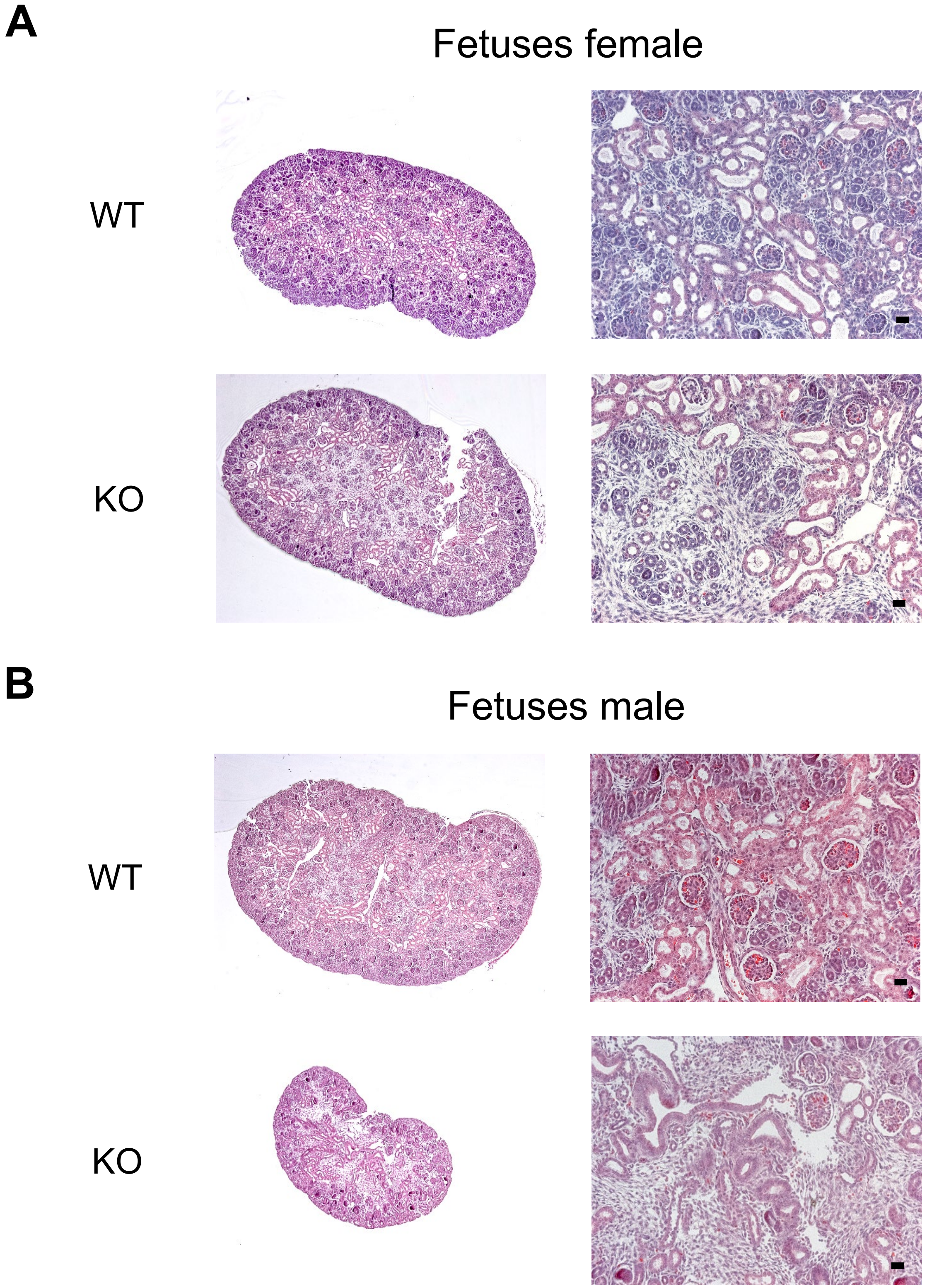

Figure S4. Status of fetal kidneys: HE staining of fetal kidney of fetuses isolated at day 18.5 from both female (A) and male (B) animals show signs of inflammation. Scale bar shows 25µm.Pala
